# Silver-loaded poly(vinyl alcohol)/polycaprolactone polymer scaffold as a biocompatible antibacterial system

**DOI:** 10.1038/s41598-024-61567-5

**Published:** 2024-05-15

**Authors:** Zuzana Vilamová, Zuzana Šimonová, Jiří Bednář, Petr Mikeš, Miroslav Cieslar, Ladislav Svoboda, Richard Dvorský, Kateřina Rosenbergová, Gabriela Kratošová

**Affiliations:** 1 Nanotechnology Centre, CEET, VSB-Technical University of Ostrava, 17. listopadu 15/2172, Poruba, 708 00 Ostrava, Czech Republic; 2 Faculty of Materials Science and Technology, FMT, VSB-Technical University of Ostrava, 17. listopadu 2172/15, Poruba, 708 00 Ostrava, Czech Republic; 3 Faculty of Material Science and Technology, Center for Advanced Innovation Technologies, VSB-Technical University of Ostrava, Ostrava, 17. listopadu 15/2172, 708 00 Ostrava, Czech Republic; 4 Department of Physics, Faculty of Science, Humanities and Education, Technical University of Liberec, Studentská 5, 461 17 Liberec, Czech Republic; 5 Department of Physics of Materials, Faculty of Mathemathics and Physics, Charles University, Ke Karlovu 3, 121 16 Prague, Czech Republic; 6 National Institute for Nuclear, Biological and Chemical Protection, V.V.I., Kamenná 71, 262 31 Milín, Czech Republic

**Keywords:** Silver nanoparticle, Electro-spraying, Antimicrobial properties, Cytotoxicity, Wound healing, Biomaterials, Characterization and analytical techniques

## Abstract

A chronic nonhealing wound poses a significant risk for infection and subsequent health complications, potentially endangering the patient‘s well-being. Therefore, effective wound dressings must meet several crucial criteria, including: (1) eliminating bacterial pathogen growth within the wound, (2) forming a barrier against airborne microbes, (3) promoting cell proliferation, (4) facilitating tissue repair. In this study, we synthesized 8 ± 3 nm Ag NP with maleic acid and incorporated them into an electrospun polycaprolactone (PCL) matrix with 1.6 and 3.4 µm fiber sizes. The Ag NPs were anchored to the matrix via electrospraying water-soluble poly(vinyl) alcohol (PVA), reducing the average sphere size from 750 to 610 nm in the presence of Ag NPs. Increasing the electrospraying time of Ag NP-treated PVA spheres demonstrated a more pronounced antibacterial effect. The resultant silver-based material exhibited 100% inhibition of gram-negative *Escherichia coli* and gram-positive *Staphylococcus aureus* growth within 6 h while showing non-cytotoxic effects on the Vero cell line. We mainly discuss the preparation method aspects of the membrane, its antibacterial properties, and cytotoxicity, suggesting that combining these processes holds promise for various medical applications.

## Introduction

Skin abrasions and cuts are everyday experiences in one’s lifetime, often resolving without complications. However, complications such as infection can form chronic, non-healing, and hard-to-heal wounds^1^. Traditional signs of inflammation, such as pain, heat, and redness, emerge as the body fights off infection. These indicators may also be accompanied by systemic pyrexia, unpleasant odor, and impaired function. As a result, chronic wounds have consequences that extend beyond the individual, impacting both patients and their loved ones, resulting in financial burdens, social withdrawal, reduced self-assurance, and potentially evoking emotions of frustration and sadness^1,2^.

Wound healing is a complex process that can depend on several factors, including appropriate dressings^1,3^. For example, it is first necessary to acknowledge acute or chronic, wet or dry, infected or non-infected wound type before choosing the dressing material specifically designed for this purpose: (1) while dressings with high water permeability are applied to wet chronic wounds, their high porosity makes them unsuitable for forming a mechanical barrier against airborne bacteria, (2) dressings with different antibacterial and antiseptic agents are used because bacterial resistance can develop during healing^3–5^. Nowadays, multilayered wound dressings^1,4^ are developed that typically consist of antibacterial agents^5,6^ and fibrous matrices^5,7,8^, providing a hygroscopic adhesive layer for new cell generation. All these materials have been seriously investigated because they accelerate wound healing and protect it from airborne bacteria and other pathogens^3,7^.

Silver dressings are used for wounds where infection is already established or excessive wound bioburden delays healing due to critical colonization or pre-infection. They are used for a short period before re-evaluation. Antibiotics generally have a single target site, and bacterial cells can more easily develop resistance. Thus, new antimicrobial drugs are developed. Moreover, there is evidence of synergism between silver and antibiotics or analgesics^9,10^.

This paper investigates Ag nanoparticles (NP) for their antimicrobial activity and proven Ag^+^ ion release^11^. The rate of this release depends on the size, shape, capping agent, and membrane adhesion of the NP^8,12,13^. NPs can induce various forms of damage to bacterial cells, including: (1) Ag NP aggregation and its adsorption on the surface of the bacterial cell, leading to membrane damage and decreased transport capability; (2) penetration of Ag NPs and ions into cellular organelles and biomolecules, altering cellular functions such as permeability and respiration; (3) generation of reactive oxygen species within the cell, resulting in cell damage; and (4) interaction of Ag NPs with bacterial DNA, leading to loss of transcription and replication^1,8,11,12,14^. The total amount of silver in the dressings varies considerably, but a partial proportion of the silver that reaches the wound site is involved in antimicrobial action; most remains in the dressing or binds to proteins in the wound and wound debris. The use of Ag products is limited to 6 weeks, and treatment should then be reassessed for possible allergic reactions. Silver dressings occasionally cause local skin discoloration or staining that is harmless and generally reversible^15,16^.

Nanofibers mats can be prepared by electrospinning method and applied in several fields including filtration^17^, drug delivery^5^, nanosensors^18^ and wound dressing as well^1,5^. Electrospinning enables the production of fibers from a wide range of polymers^19,20^ or polymers blends with antibacterial agents^1,5^ while allowing control over the properties of the nanofiber mat, such as the fiber’s size distribution, porosity, or mat thickness^19^. The main advantages of using electro-spraying in medicine include producing bioactive agents such as NPs ^21^ and droplets, which act as drug carriers^4,22,23^. Encapsulation, documented in scientific literature, involves a straightforward droplet preparation process: (1) homogeneously dissolve bio-agents in a polymer solution through stirring or ultrasonication, followed by easy electro-spraying, or (2) allow bio-agents to interact with droplets in the particle collection bath, facilitating cross-linking and the formation of new bonds^4,23^.

Materials used for electrospinning and electro-spraying in the medical field include water-soluble synthetic polymers such as PVA, polyvinyl pyrrolidone, and polyethylene glycol, and water-insoluble synthetic polymers including PCL, polyacrylonitrile, polyvinylidene fluoride and polyurethane. These materials are then used as carriers for bioactive agents, such as NPs^1,24^, or as templates or artificial fibers to form fibrous products with complex and unique 3D structures^25,26^. Biodegradable synthetic polymers like PVA, PCL, polyglycolic acid, and polylactic acid offer numerous medical advantages. They facilitate the creation of electro-spun 3D-fiber structures that closely mimic the natural extracellular matrix, essential for optimal cell attachment and growth ^1,4^.

PVA is an odorless crystalline water-soluble polymer widely used in medical applications because it has high biocompatibility, low toxicity, and low tendency for protein adhesion^27^. In contrast, PCL is a semi-crystalline hydrophobic polymer. Therefore, it is water-insoluble, bio-degraded in human physiological conditions, and has proven biocompatibility. PCL polymer is also widely investigated for its physical, thermal, and mechanical properties, which depend mainly on its 3000–80,000 g/mol molecular weight, its degree of crystallinity, and its formation, such as PCL mats or fibers^28,29^. Moreover, the electro-spun materials are increasingly investigated because of their structural similarity to the dermal extra-cellular matrix and easier cell adhesion^25,26^.

We, therefore, prepared a polymer-metal-based membrane using electrostatic processes. As a water-soluble polymer, PVA served as an Ag NPs carrier, which dissolved after contact with humidity and caused a burst of Ag NPs released. After the proven antibacterial effect of adsorbed Ag NPs in the wound, PCL should serve as a matrix for cell growth. In this paper, we focused on the following: (1) Ag NP preparation by the simple chemical method, (2) PCL electrospinning, and (3) electro-spraying Ag NPs in a PVA polymer-blend solution on a PCL matrix. The final materials’ G+ *S. aureus* and G− *E. coli* antibacterial properties were then investigated. Finally, awareness of possible Ag NPs cytotoxicity led to our investigation of their effect on the Vero cell line.

## Results and discussion

### Characterization of Ag NPs

**Figure 1:**
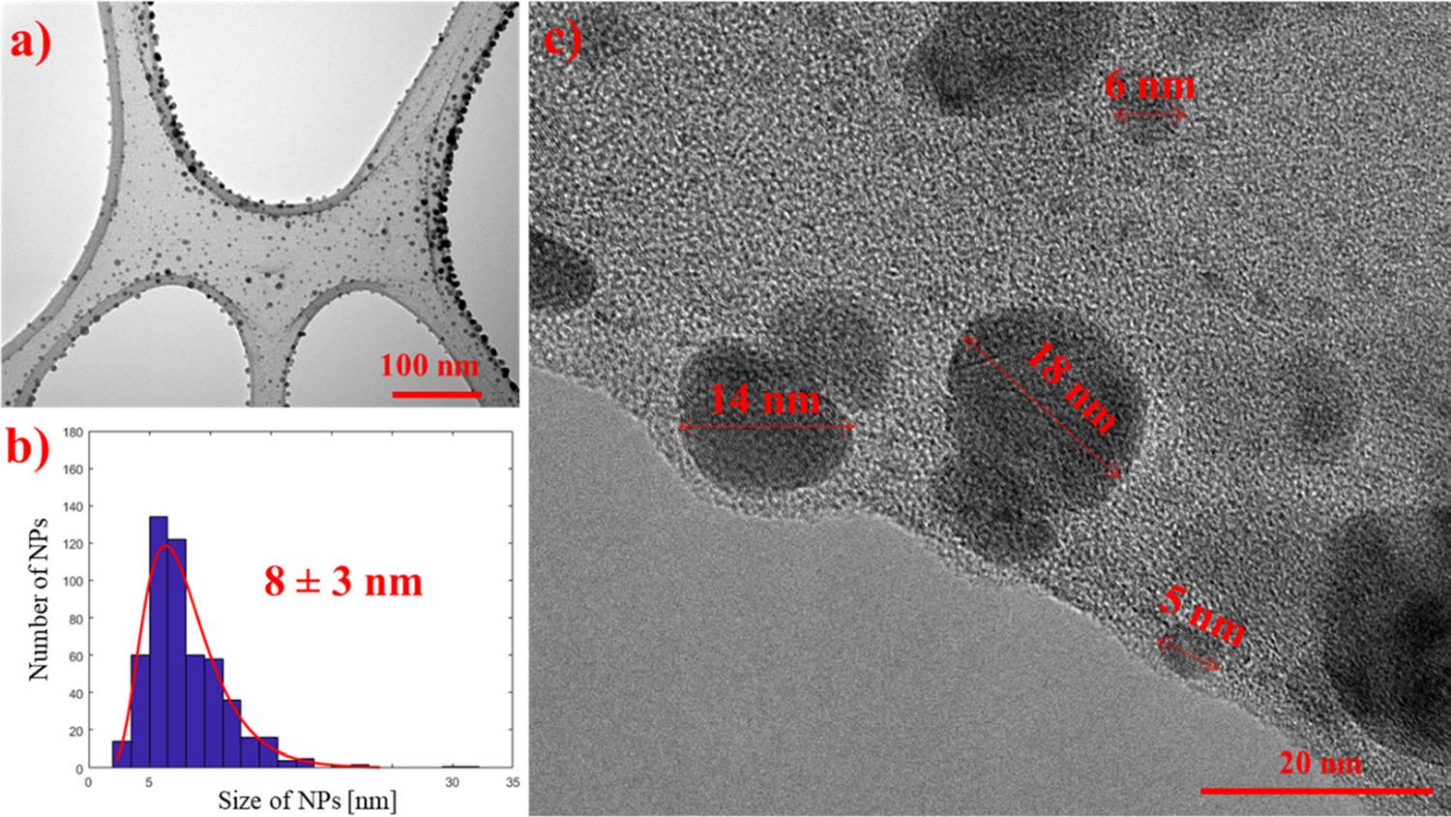
TEM image of (**a**) Ag NPs; scale bar 100 nm, with (**b**) their size distribution 8 ± 3 nm, and (**c**) HRTEM NPs size analysis; scale bar 20 nm.

The successfully prepared Ag NPs were mostly spherical and oval–polygonal in shape, and this was confirmed by HRTEM analysis (Fig. 1a). Figure 1b then depicts the 8 ± 3 nm mean core size, and Fig. 1c shows the NPs under 20 nm.

**Figure 2:**
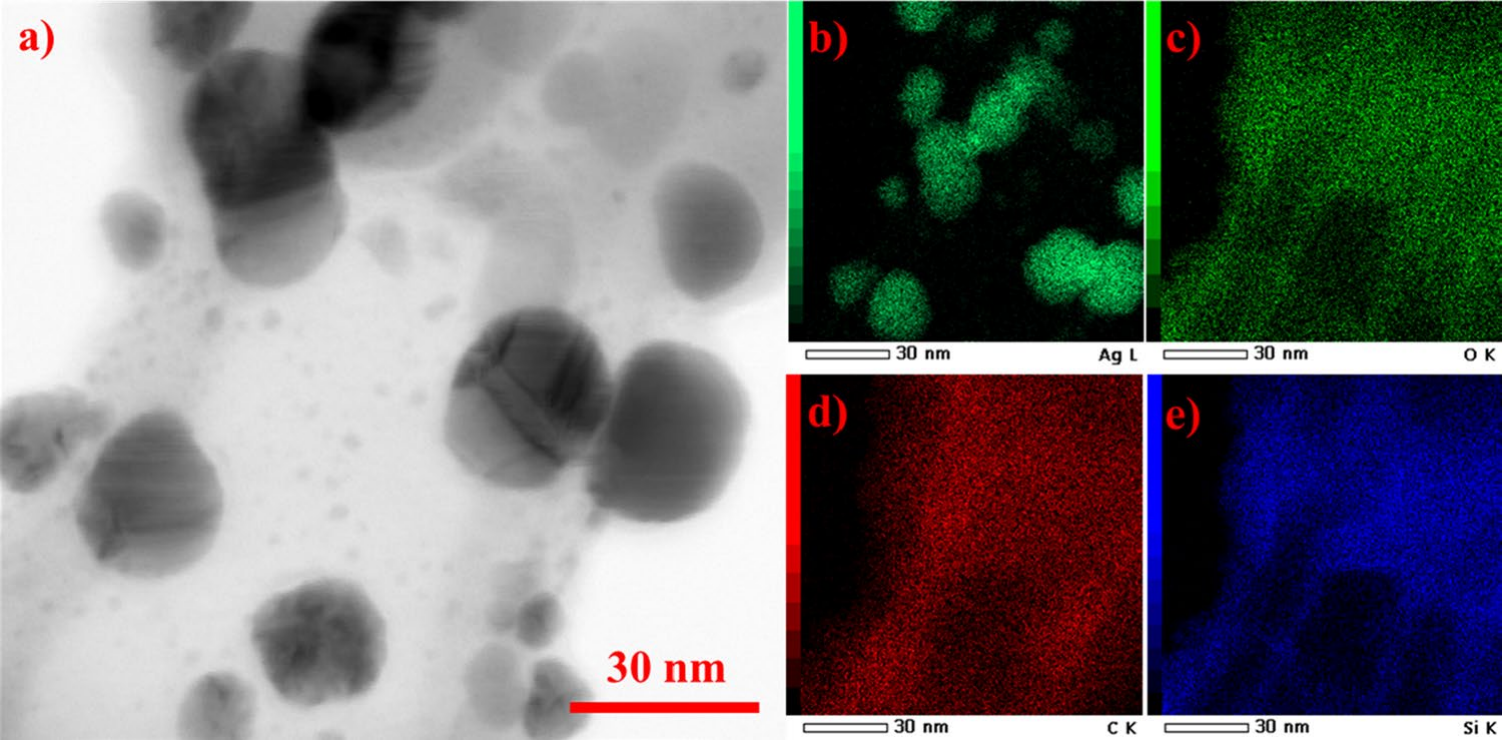
(**a**) STEM bright field (BF) and EDS mapping of prepared (**b**) Ag NPs with other elements (**c**) O, (**d**) C and (**e**) Si present of carbon/formvar grid used for HRTEM/STEM analysis; scale bar 30 nm.

The characteristic interplanar distances and main lattice planes of FCC Ag NPs are as follows: 2.359 Å for (111), 2.044 Å for (200), 1.445 Å for (220) and 1.231 Å (311)^24,30^. Our HRTEM FFT Ag NP analysis identified the main planes of FCC Ag NPs, specifically (111) and (200) (see Supplementary Figure S1 and Table S1). However, XRD analysis (Supplementary Figure S2) did not exhibit the prominent peaks corresponding to cubic Ag NPs forms, such as (111), (200), and (220), which typically appear at around 38°, 44° and, 64° on the 2θ scale^24^. Nevertheless, this could be attributed to the notable presence of residue from silver nitrate, confirmed by XRD analysis and visible in the TEM image provided in Supplementary Figure S2. Furthermore, the EDX mapping analysis depicted in Fig. 2 provides further evidence confirming the presence of Ag NPs synthesized through MA and silver nitrate (AgNO_3_).

### Characterization of fibrous matrixes

The decrease in overall polymer viscosity value from 17.08 ± 0.1 to 13.92 ± 0.1 mPa·s is most likely attributed to the presence of Ag NPs. This decrease is supported in the study^24^ and is elaborated upon by Chou et al.’s molecular modeling of the interaction between Ag NPs and PVA^31^. The interaction between Ag NPs and the −OH groups of PVA may reduce the extent of hydrogen bond formation among individual PVA molecules, consequently resulting in decreased viscosity of the PVA+Ag mixture. A decrease in the viscosity of polymer solutions such as PVDF^32^ was also observed upon adding Ag NPs. Moreover, the viscosity values are influenced by polymer Mw, mass concentration, filler, solvent, and measuring apparatus. It is also possible that other compounds present in the colloid, such as AgNO_3_ and an acidic environment, influence the final viscosity^33^.

**Figure 3:**
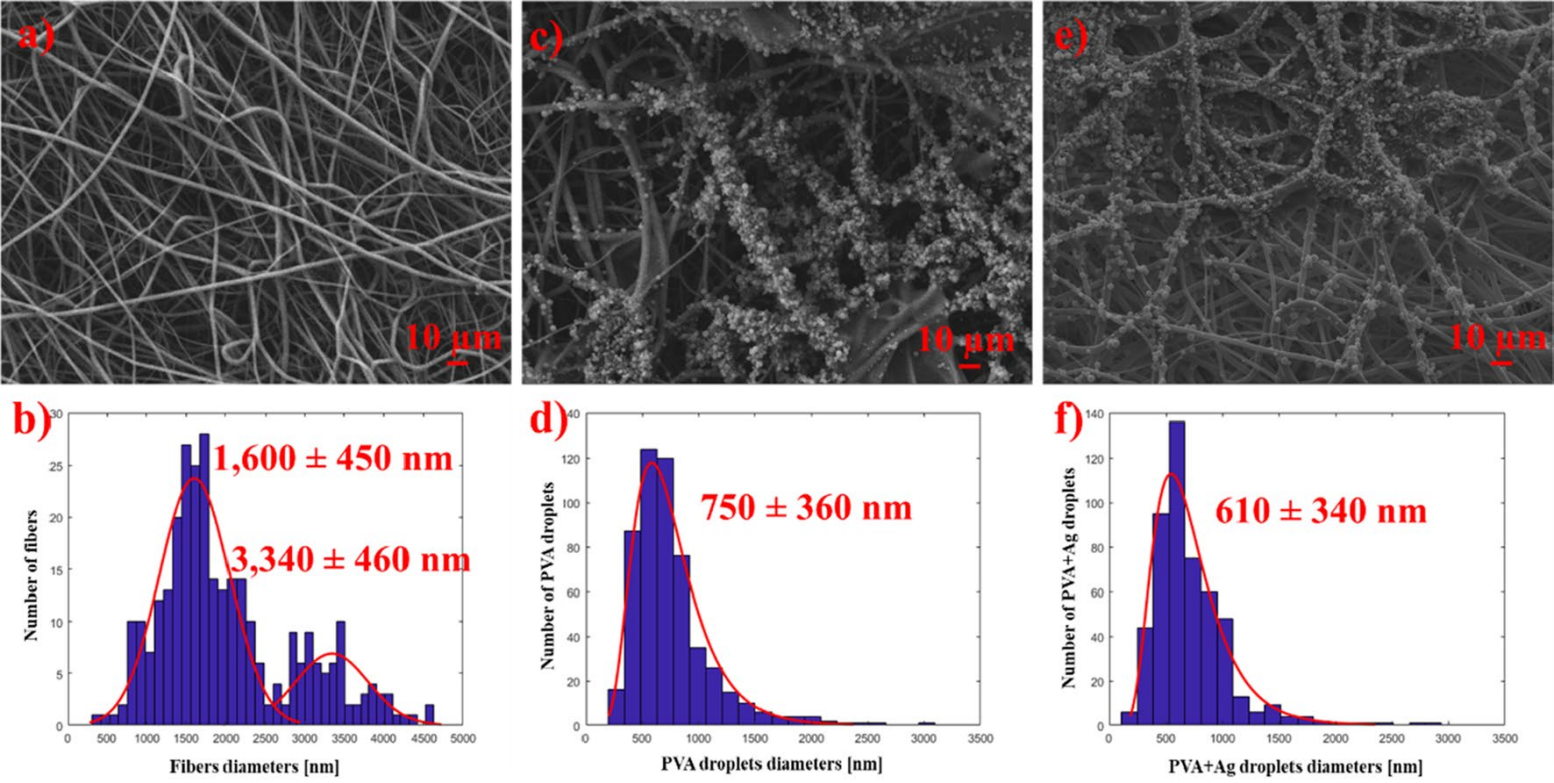
SEM images of (**a**) pure PCL fibers with its (**b**) bimodal fiber size distribution, (**c**) PCL/PVA_60 with (**d**) PVA droplets size distribution, and (**e**) PCL/PVA + Ag_60 with (**f**) PVA+Ag droplets size distribution; scale bar 10 µm.

Figure 3a demonstrates the SEM analysis of PCL fibers, confirming their successful preparation. The bimodal diameter distribution is illustrated in Fig. 3b. In the pure PCL matrix, the most frequently observed fiber diameters were approximately 1700 nm.

While PVA droplets, see Fig. 3c, exhibited an average diameter of 750 ± 360 nm (Fig. 3d), with 685 nm being the most common diameter, PVA+Ag droplets, Fig. 3e, had an average diameter of 610 ± 340 nm (Fig. 3f), with approximately 610 nm being the most frequently observed. Consequently, the PVA+Ag droplets were smaller in diameter compared to the pure PVA droplets. Furthermore, the decrease in viscosity induced by Ag NPs could result in the observed reduction in average droplet size, as reported in another study focusing on PVA fibers^24^. Finally, huge droplets over 1500 nm were also occasionally noted.

**Figure 4:**
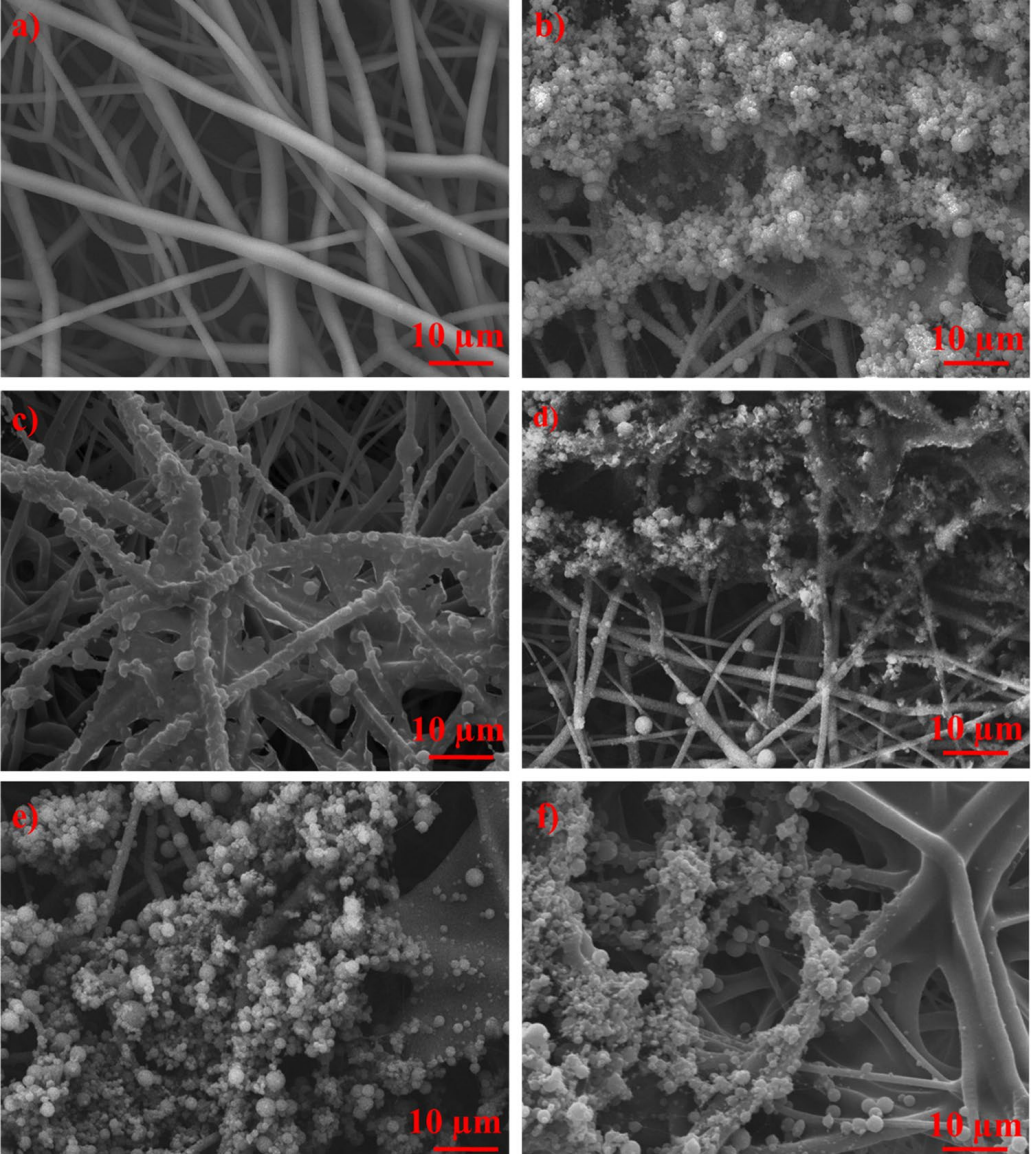
SEM images of prepared samples: (**a**) pure PCL fibers and (**b**) PCL/PVA_60 with droplets on the surface and in the first PCL fiber layer. The Ag NPs prepared from 0.1 mM MA were encapsulated in PVA droplets and applied for different times: (**c**) 15, (**d**) 30, (**e**) 45, and (**f**) 60 min; scale bar 10 µm.

Figure 4a shows the smooth surface of the pure PCL fibers, contrasting with the samples with electro-sprayed PVA (Fig. 4b) and PVA+Ag droplets (Fig. 4c, d). The electro-spraying duration played a crucial role in the accumulation of PVA+Ag droplets on the fiber surface, within, and beneath the outer layer of the first fibrous material. The quantity of PVA+Ag droplets adhered to the PCL fibrous matrix varied with electro-spraying durations of 15, 30, 45, or 60 min (Fig. 4c–f). Importantly, the number of attached PVA+Ag droplets increased with longer electro-spraying durations, leading to the highest accumulation of PVA+Ag droplets for the PCL/PVA+Ag_60 sample.

**Figure 5:**
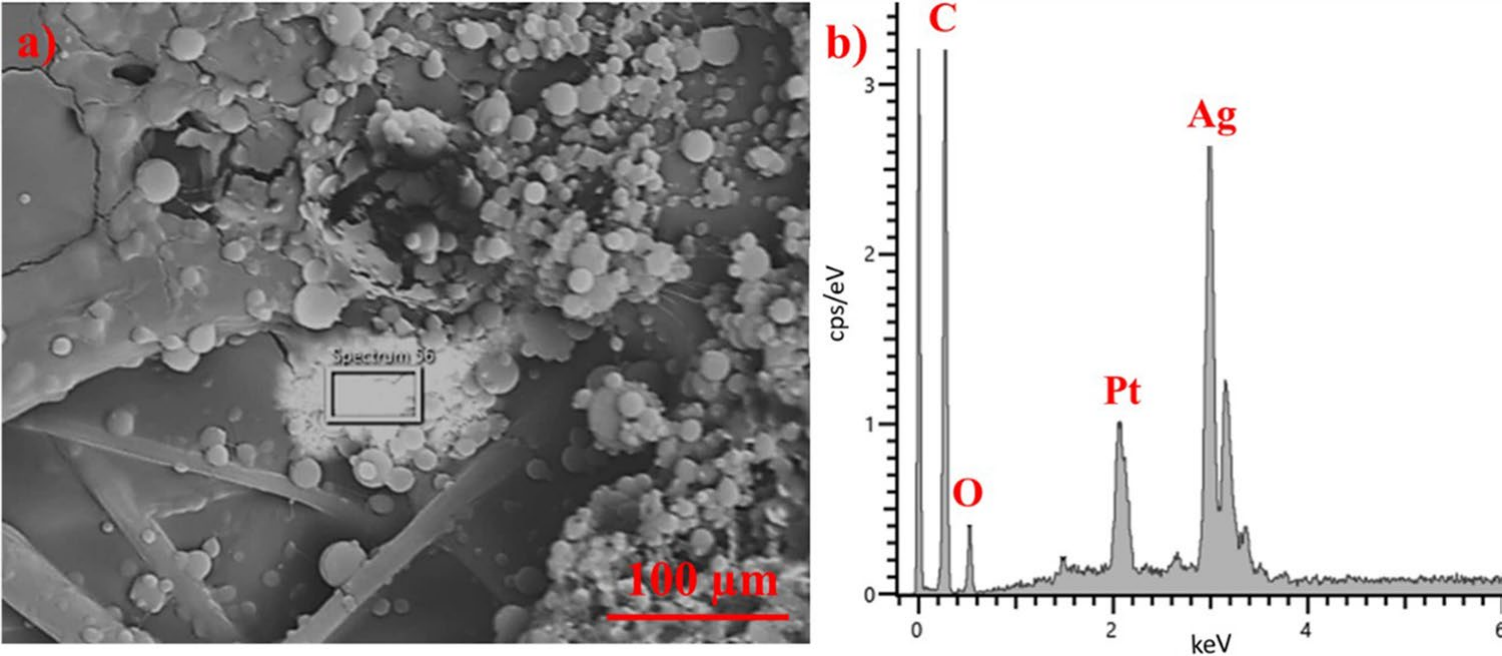
SEM image of (**a**) the sample PCL/PVA+Ag_45 with droplets, PCL fibers, and location of Ag (79.7 ± 0.5 wt%) and b) EDX analysis of selected area; scale bar 100 µm.

EDS analysis was utilized to confirm the presence of Ag. The random clusters of Ag, constituting 79.7 ± 0.5 wt%, in Fig. 5a, were likely a result of electro-spraying parameters such as high voltage and the non-uniformity of droplets trapped on the static collector. Additionally, the carbon content of 15.2 ± 0.4 wt% and oxygen content of 4.7 ± 0.3 wt% were attributed to the structures of PVA and PCL, respectively. The presence of platinum was observed because the samples sputtered for SEM analysis.

### Antimicrobial evaluation

G+ *S. aureus* and G− *E. coli* are frequently employed bacterial strains. Additionally, these strains have developed antibiotic resistance, highlighting the urgent need for alternative treatments for bacterial infections^5,7,34^.

Tables 1 and 2 highlight the demonstrated effectiveness of Ag NPs attached to a fibrous matrix against *S. aureus* and *E. coli*. Despite the few droplets containing Ag NPs observed in the 15 min electro-spraying sample, even this brief duration exhibited antibacterial activity against *S. aureus* after 24 h. However, *E. coli* growth was not inhibited during testing time for the PCL/PVA+Ag_15 sample. The PCL/PVA+Ag_30 and PCL/PVA+Ag_45 samples demonstrated antibacterial activity against both bacterial strains after 24 h. Notably, a more rapid antibacterial effect was observed against G+ *S. aureus* compared to G− *E. coli*, with growth inhibition observed after 3 h for these samples. The efficiency of the PCL/PVA+Ag_60 sample was impressive, as evidenced by growth inhibition of both bacterial species after 3 h of incubation, reaching 100% inhibition after 6 h. As expected, control samples consisting of pure PCL and PCL/PVA_60 without Ag NPs provided no antibacterial effect against either bacterium.

**Table 1﻿:** Antibacterial evaluation of prepared control fibrous samples and samples with Ag NPs against *S. aureus*.

Incubation time	2 h	3 h	6 h	12 h	24 h
Sample	Growth inhibition (100%)				
PCL fibers	**Negative**	**Negative**	**Negative**	**Negative**	**Negative**
PCL/PVA_60	**Negative**	**Negative**	**Negative**	**Negative**	**Negative**
PCL_PVA+Ag_15	**Negative**	**Negative**	**Negative**	*Slowing growth*	***Positive***
PCL_PVA+Ag_30	**Negative**	*Slowing growth*	***Positive***		
PCL_PVA+Ag_45	**Negative**	*Slowing growth*	***Positive***		
PCL_PVA+Ag_60	**Negative**	*Slowing growth*	***Positive***		

***Positive*** means total bacterial inhibition, *Slowing growth* inhibition means bacterial colonies between 10^1^–10^4^/mL, and **Negative** means any inhibition observed.

**Table 2:** Antibacterial evaluation of prepared control fibrous samples and samples with Ag NPs against *E. coli*.

Incubation time	2 h	3 h	6 h	12 h	24 h
Sample	Growth inhibition (100%)				
PCL fibers	**Negative**	**Negative**	**Negative**	**Negative**	**Negative**
PCL/PVA_60	**Negative**	**Negative**	**Negative**	**Negative**	**Negative**
PCL_PVA+Ag_15	**Negative**	**Negative**	**Negative**	**Negative**	**Negative**
PCL_PVA+Ag_30	**Negative**	**Negative**	**Negative**	*Slowing growth*	***Positive***
PCL_PVA+Ag_45	**Negative**	**Negative**	**Negative**	*Slowing growth*	***Positive***
PCL_PVA+Ag_60	**Negative**	*Slowing growth*	***Positive***		

***Positive*** means total bacterial inhibition, *Slowing growth* inhibition means bacterial colonies between 10^1^–10^4^/mL, and **Negative** means any inhibition observed.

Our results generally indicated that samples containing Ag NPs exhibited stronger antibacterial activity against G+ *S. aureus* bacteria than G− *E. coli*, which could be attributed to their structural differences. Firstly, the G− bacteria have a cell wall consisting of an outer lipopolysaccharide membrane and an inner peptidoglycan membrane, which is relatively thin and only 3–4 nm thick. In contrast, G+ bacteria lack an outer lipopolysaccharide membrane, but their peptidoglycan layer is approximately 30 nm thick. These structural distinctions, particularly in the thickness and composition of the cell wall, might elucidate why G+ *S. aureus* is less susceptible to Ag NPs compared to G− *E. coli*. Furthermore, this observation aligns with findings from other studies investigating the antibacterial activity of Ag NPs^1,7,12,35,36^.

The antibacterial properties of Ag NPs are significantly influenced by their size^8,12,13^. Higher antibacterial activity is observed with smaller Ag NPs^13^, as they can easily penetrate through bacterial cells and interact with DNA, leading to increased production of reactive oxygen species and subsequent damage to bacterial cells^8,12–14^.

Bacteria such as *E. coli* have a 2 µm rod length and 1.1–1.5 µm radius/width, while *S. aureus* is round with 0.5–1 µm size^37^*.* A particle permeability test was conducted as part of our assessment of the material’s mechanical and antibacterial properties. This test utilized spherical model particles with a size distribution of 20–1000 nm, and their capture activity was subsequently evaluated. Notably, this range of 20–1000 nm is smaller than the size of bacterial cells. The pure PCL_PVA_60, PCL_PVA+Ag_15, PCL_PVA+Ag_30, PCL_PVA+Ag_45 and PCL_PVA+Ag_60 fibers were measured with the following membrane efficiency values: 81.8, 91.7, 79.6, 71.4, 76.7 and 86.5 cm/s, and the air permeability was 27.5, 13.7, 22.3, 27.1, 27.6 and 16.4 m/s according to EN 14683:2019+AC. The membrane was concurrently permeable to air, and most particles were captured. Some minor variations were observed among the samples; although specific samples appeared thicker than others, the surface remained consistent during measurements. Differences may also result from the random capturing of PCL fibers during the electrospinning process. Notably, the highest concentration of fibers tends to occur in the middle of the collector, resulting in less capture at the edges^38^. In conclusion, the results prompted us to conclude that all membranes serve as mechanical barriers against airborne bacteria.

Although Ag is an acknowledged antibacterial agent against both G+ and G− bacteria, the effects of Ag NP dressings vary depending on the specific Ag species released and the substrate utilized^39^. Moreover, materials treated with Ag NPs and other Ag compounds are usually applied for only 4–6 weeks in medical practice because of the developing rapid resistance of some bacterial strains after repeated exposure^3,15,40,41^.

### Cytotoxic evaluation

It is, therefore, necessary to consider the risks and benefits of different Ag dressings for appropriate wound healing. As Ag NPs exhibit antibacterial efficiency, they can exhibit cytotoxic effects on mammalian cells at certain concentrations^8,11,12^. This cytotoxicity must also be investigated. The PCL/PVA+Ag_60 sample demonstrated the highest antibacterial activity against both tested bacteria cultures. Hence, it was chosen as the representative sample for cytotoxicity testing. Furthermore, the release kinetics of Ag from the sample were also examined, and the details can be found in Supplementary Figure S3.

**Figure 6:**
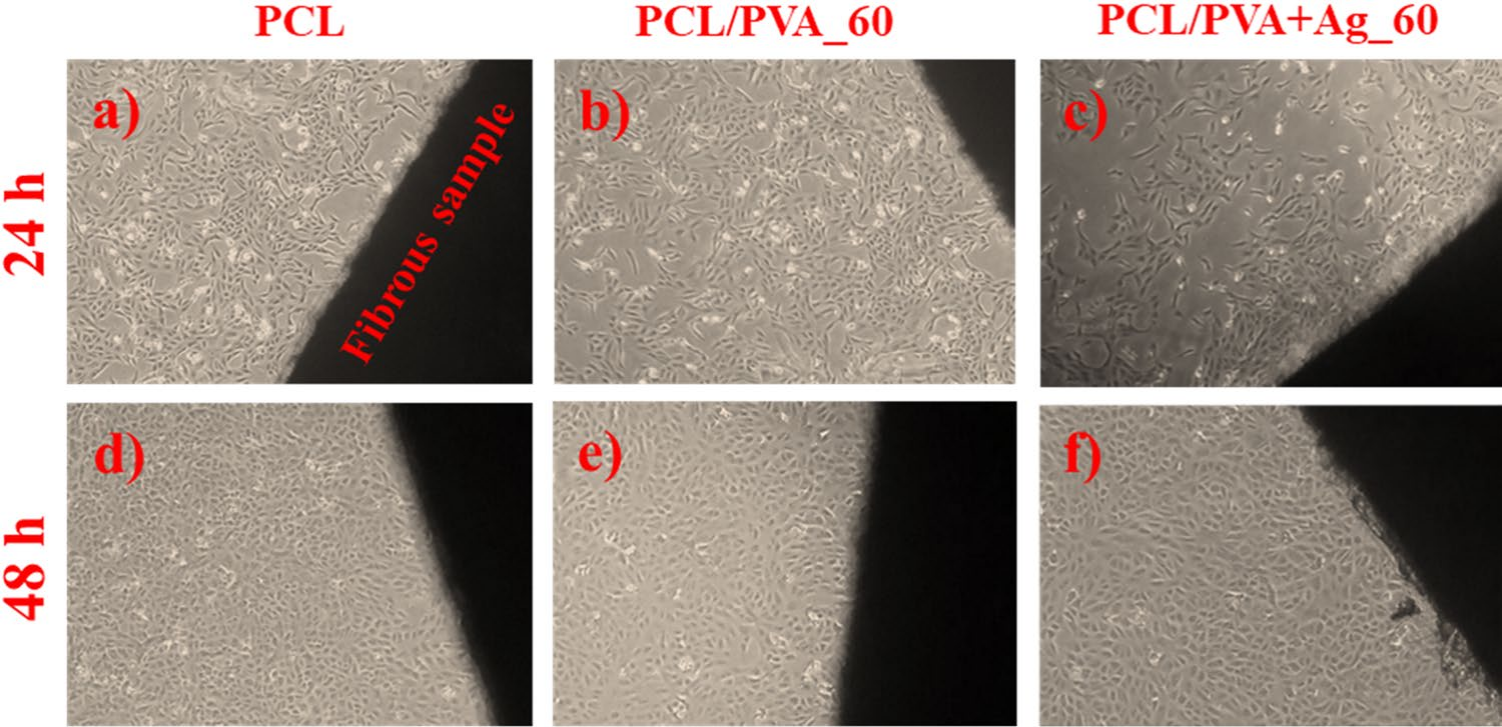
Light microscope images of direct cytotoxicity testing after 24 h; (**a**) PCL fibers (control), (**b**) PCL/PVA_60 (control), (**c**) PCL/PVA+Ag_60; and 48 h; (**d**) PCL fibers (control), (**e**) PCL/PVA_60 (control), (**f**) PCL/PVA+Ag_60; magnification ×50.

Figure 6 depicts the direct testing of samples, including the pure PCL fibrous control, the PCL/PVA_60 fibrous control, and the experimental PCL/PVA+Ag_60. No morphology or cell growth alterations were observed in these samples following 24- and 48 h incubation periods. Evaluation according to the ČSN EN ISO 10993-5 cytotoxicity guidelines for direct contact testing indicated that the samples were non-cytotoxic at stage zero. The life cycle of the cells in contact with this fibrous material was, therefore, not disturbed in any way. Moreover, the tested surface did not affect cell physical–chemical bonding, and the cells could still replicate (Fig. 6).

The MTT assay is the most common method for testing cell growth rate and culture medium toxicity. This assay measures cell viability, wherein cells reduce yellow water-soluble MTT to blue/violet insoluble formazan crystals, indicating cellular activity. The intensity of color, determined through photometric measurements after dissolving the formazan crystals in alcohol, correlates with the number of viable cells. If the viability of the sample drops below 70% of the control, it indicates cytotoxic potential^12,35,42,43^.

Decreasing extract concentration determined control cell viability; PC viability values were 0, 1, 59, and 82%, and NC values were 97, 96, 98, and 99%.

**Figure 7:**
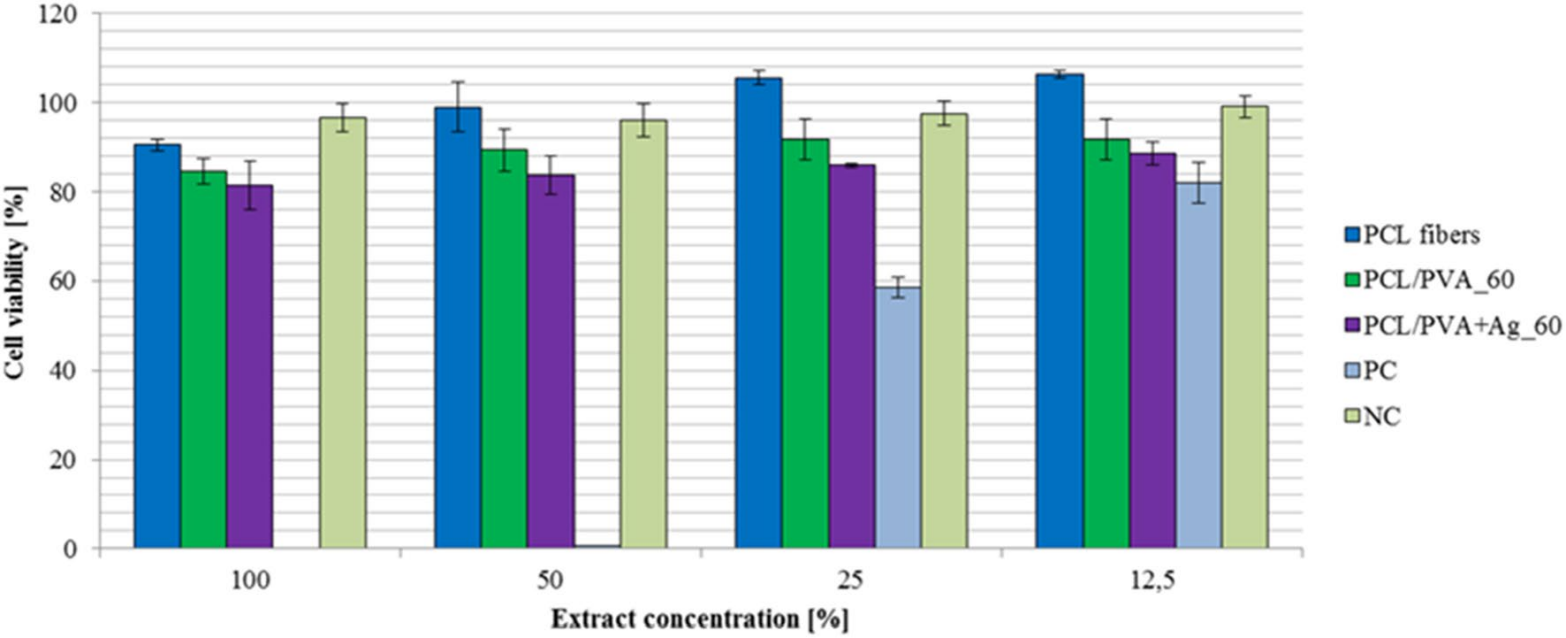
Viability of Vero cell suspension with error bars in the presence of samples: PCL fibers, PCL/PVA_60, PCL/PVA+Ag_60, and PC and NC control samples. Presence of samples in left to proper color order: PCL fibers, PCL/PVA_60, PCL/PVA+Ag_60, positive control (PC), and negative control (NC).

Cell viability was evaluated at decreasing 100, 50, 25, and 12.5% extract concentrations as can be seen in Fig. 7; (1) PCL fibers established 91, 99, 106, and 106% cell viability; (2) the PCL/PVA sample enabled 90, 89, 92 and 92% cell viability and (3) the PCL/PVA+Ag_60 sample exhibited 82, 84, 86 and 89%. Figure 7 highlights that the results indicate non-toxicity of all samples to cells. The findings suggest that pure PCL fibers serve as viable scaffolds for tissue engineering, given the high cell viability observed. Other studies corroborate this assertion^25,26,44^.

## Materials and methods

### Materials preparation

The Ag NPs were synthesized using maleic acid (MA, C_4_H_4_O_4_, Mw = 116.07 g/mol, ≥ 99% purity, Carl Roth, Germany) and silver nitrate (AgNO_3_, M_w_ = 169.88 g/mol, ≥ 99%, Carl Roth, Germany). The 10 mmol/dm^3^ AgNO_3_ precursor was added in 1:1 ratio to 0.1 mmol/dm^3^ MA solution, and the final colloid was kept at 4 °C in the dark for further use.

A 10 wt% PCL solution was prepared (M_w_ = 80,000 g/mol, Sigma–Aldrich, USA), and this was stirred for 24 h in a 4:1 chloroform/ethanol solution to obtain a liquid solution. The fibrous matrix was prepared by Nanospider^™^ NS 1WS500U (Elmarco, Czech Republic). The experiment was performed at 22.9 °C and 36.5% humidity, with 16 cm between the 10 kV negatively charged collector and the 50 kV positive electrode. The PCL fibers were then formed on a spun-bond at 14 mm/min substrate-shift.

The following two polymer solutions were prepared before the electro-spraying process: (1) A 10 wt% solution of solid PVA (PVA, M_w_ = 9000–10,000 g/mol, 80% hydrolyzed, Sigma–Aldrich, USA) was diluted in DEMI water and (2) 10 wt% solution of solid PVA from the same producer was diluted in prepared Ag colloid (PVA+Ag) at 23 °C room temperature and stirred for 2 h. The electro-spraying was performed by a 4SPIN^®^ device (Contipro, Czech Republic) using a Ø = 0.8 mm stainless steel needle and the C1 static collector covered with previously prepared PCL fibers. Samples sprayed on the PCL matrix were obtained at an optimized 25 kV applied voltage, 10 µl/min flow rate, and 18 cm collector-emitter distance. The PVA control samples were sprayed for 60 min and labeled PCL/PVA_60, and the PVA+Ag materials were prepared by 15, 30, 45, and 60 min spraying to obtain gradually increased Ag NP content in the PVA droplets. Prepared PCL matrices with PVA+Ag were designated according to the following spraying times: PCL/PVA+Ag_15; PCL/PVA+Ag_30; PCL/PVA+Ag_45 and PCL/PVA+Ag_60. All experiments were conducted at 23 °C laboratory temperature and approximately 36% humidity.

### Characterization methods

The Ag NP’s morphology and size were observed under JEOL 2200FS high-resolution transmission electron microscope (HRTEM/STEM) with Centurio EDS detector (JEOL, Japan) and JEOL 1011 transmission electron microscope (TEM, JEOL, Japan). 2 μl samples were transferred onto carbon/formvar grids. TEM images were post-processed by JMicroVision software, and a minimum of 500 Ag NPs were evaluated to obtain size distribution parameters. Finally, fast Fourier transformation (FFT) was applied in ImageJ software (National Institute of Health, USA) for HRTEM analysis.

A volume of 100 μL of the colloidal sample was applied five times onto a microscope slide and then dried at 50 °C for 30 min to produce a representative thin layer of the sample on a glass plate. X-ray diffraction (XRD) patterns were acquired using a Bruker D8 DISCOVER diffractometer (Bruker AXS, Billerica, USA) equipped with an X-ray tube featuring a rotating Cu anode with a wavelength (λ) of 1.5418 Å and a power of 12 kW. All measurements were conducted in parallel beam geometry with a parabolic Goebel mirror in the primary beam. The X-ray diffraction patterns were ultimately recorded in grazing incidence over a 2θ range from 5 to 80°, employing a step size of 0.05° and an angle of incidence (α) of 1.5°.

The viscosity of the homogeneous PVA and PVA+Ag solutions was measured by DV2T Viscometer (Brookfield Ametek, USA), and the dynamic viscosity was determined from the magnitude of the torque (above 70%) measured in steady-state rotation at appropriate SC4-18 spindle speeds in the SC-13R sample container.

The PVA droplets and PCL fibrous samples were identified by scanning-transmission electron microscopy (STEM, JEOL JSM-7610F+JEOL, Japan) at 15 kV applied voltage. The samples were sputtered with a thin 20 nm layer of platinum, and the morphology and element composition analyses were performed by STEM equipped with energy-dispersive X-ray spectroscopy (EDS) and Aztec Ultima Max 65 microanalyzer (Oxford Instruments, UK). The electro-spun fiber size distribution was then evaluated by image analysis (JMicroVision software). We analyzed over 250 fiber and 500 droplet diameters from the STEM micrographs.

The Ciros Vision ICP-AES spectrometer (SPECTRO Analytical Instruments Inc., Kleve, Germany) was used to determine Ag released from fibrous samples. Approx. 2 × 2 cm of PCL/PVA+Ag_60 sample was added to DEMI water of 37 °C. After 60 min, the total Ag content of the tested sample was measured and fit $y=a\bullet (1-{e}^{-bx})$.

Membrane filter efficiency was measured by our assembled laboratory device according to the EN 14683:2019+AC European standard. Standard HTR-B tubes with 50 mm outer diameter were used as filter holders with 17 cm^2^ defined area (Gebr. Ostendorf Kunststoffe GmbH, Germany). Panel mounting flowmeter LZM-15Z was used (0.6–6 m^3^/h, with ± 4% accuracy, Thermis, Czech Republic) and the airflow was regulated by Bosch EasyVac 3 turbine (Bosch, Czech Republic) with 19.7 cm/s LZM-15Z flowmeter speed attained. PortaCount Pro 8030 (TSI Incorporated^™^, USA) with 10% factor-accuracy fit then measured the filtration efficiency by particle counting. Environment particles were generated in PALAS AGK 2000 (Palas GmbH, Germany) with 20 wt% KCl solution, and the particle distribution was then measured by Dekati ELPI+ (Dekati Ltd., Finland).

### Antibacterial properties

The prepared materials were PCL, PCL/PVA_60, PCL/PVA+Ag_15, PCL/PVA+Ag_30, PCL/PVA+Ag_45 and PCL/PVA+Ag_60. Their antibacterial activity was evaluated against G+ *S. aureus* CCM 299 and G− *E. coli* CCM3954 bacterial cultures obtained from the collection of microorganisms (Brno, Czech Republic). Fibrous materials were cut into the required square shapes and placed in Eppendorf tubes with the bacterial solutions with an initial inoculum of 10^5^/mL. The bacterial suspensions were removed after 2, 3, 6, 12, and 24 h, placed on blood agar plates, and evaluated so antibacterial testing conformed with the 100-2004 AATCC test method. Results are written in Tables 1 and 2 when positive means total bacterial inhibition, slow inhibition means bacterial colonies between 10^1^–10^4^/mL, and negative is any inhibition observed.

### Cytotoxicity activity

The PCL, PCL/PVA_60, and PCL/PVA+Ag_60 fibrous samples were cut to the required diameters and sterilized by UV lighting for three hours from each side.

Vero cell cultivation was identical for both tests (passage 44, African Monkey Kidney, ATCC, USA). The cells were cultivated in minimum essential medium with Earle’s salts (EMEM; Biowest, France) and supplemented with 10% v/v fetal bovine serum (FBS; Biowest, France). A 2 mL suspension of Vero cells was then removed from the culture flask by enzymatic digestion (trypsin/EDTA, Sigma–Aldrich, USA), and the cell suspension was centrifuged. Finally, the cells were resuspended in a culture medium at 1 × 10^5^ cells/mL density. The direct-contact test and MMT assay are performed in accordance with ČSN EN ISO 10993-5.

#### Direct-contact test

Duplicate prepared 1 × 1 cm quantities of sterile material were placed in the center of a 6-well plate and loaded with stainless-steel weights to prevent material movement. This was followed by 2 mL of prepared Vero cell suspension seeded in 6-well plates at 2 × 10^5^ cells/well plate with the samples. The cells were incubated for 24 and 48 h in an incubator with 5% CO_2_ at 37 °C and over 90% humidity. Qualitative cytotoxicity evaluation was then assessed on morphology, vacuolization, detachment, cell lysis, and membrane integrity under CKX41 Olympus optical microscopy (Olympus, Japan).

#### MTT assay

Samples were cut to the required 6 cm^2^ surface area, and a culture medium with 10% v/v FBS was used for extraction because it can support cellular growth and extract both polar and nonpolar substances. This extraction was performed 24 h at 37 °C in sterile containers and under aseptic techniques.

Here, 100 µL of Vero cell suspension was seeded into 96-well plates at 1 × 10^4^ cells in each well. The cells were incubated for 24 h in an incubator (5% CO_2_, T = 37 °C, > 90% humidity) and formed a semi-confluent monolayer. After incubation, the medium was aspirated from the cells, and 100 µL of the treatment medium was added. This contained the extract concentration with positive latex (PC) or negative polystyrene controls (NC) added to the cell culture plates. The extract concentrations were 100, 50, 25 and 12.5% v/v.

After mixing, the cells were first incubated for 24 h (5% CO_2_, T = 37 °C, > 90% humidity). The culture medium was then removed from the plates, and 50 μL of the 3-(4,5-dimethylthiazol-2-yl)-2,5-diphenyltetrazoliumbromid solution (MTT, 1 mg MTT/mL complete medium, Sigma–Aldrich, USA) was added to each well. The plates were incubated for 2 h in the incubator (5% CO_2_, T = 37 °C, > 90% humidity). Finally, the MTT solution was decanted, and 100 μL of isopropyl-alcohol was added to each well to dissolve the formazan crystals formed in the cells.

The plates were shaken for a short time using a plate shaker, and the absorbance was measured using a 570 nm filtered microplate reader. Cell viability was then calculated from the mean MTT of the three replicate values in each test concentration. This value was compared to the mean MTT value of all blank controls, and relative cell viability was finally expressed as a percentage of the control values.

## Conclusion

Silver nanoparticles ranging in size from 8 ± 3 nm were effectively synthesized by maleic acid in an aqueous environment and then fixed on a fibrous PCL matrix by electrospraying with water-soluble PVA. Several polymer-metal nanofibrous composites were prepared and, in particular, the variant containing the highest concentration of Ag (PCL/PVA+Ag_60) exhibited complete bacterial inhibition (100%) against both Gramme-positive *S. aureus* and Gramme-negative *E. coli* strains to 6 h while maintaining noncytotoxicity toward the Vero cell line.

Our process involves connection of the nanofiber and spherical polymer structure during material preparation. Subsequently, the electro-spraying process is used to effectively deliver antibacterial agents during postprocessing to the PCL matrix. Moreover, water-soluble poly(vinyl alcohol) (PVA) as an Ag NP carrier could allow the rapid release of Ag NPs into a moist environment, where its effect as an antibacterial agent was proven.

Finally, our material preparation method offers the flexibility for various modifications, including (1) preparation of a multilayered structure, where sustained release of antibacterial agent could be possible, (2) altering the morphology, size, and surface properties of active Ag NPs, and (3) increasing the concentration of Ag NPs by extending the spraying time of the PVA+Ag polymeric solution. Although these modifications enhance the antibacterial efficiency of the membrane, potential increases in cytotoxicity must be carefully monitored and addressed when feasible.

### Supplementary Information

Supplementary Information.

## Data Availability

The datasets used and/or analysed during the current study available from the corresponding author on reasonable request.

## References

[1] Sarviya N, Mahanta U, Dart A (2023). Biocompatible and antimicrobial multilayer fibrous polymeric wound dressing with optimally embedded silver nanoparticles. Appl. Surf. Sci..

[2] Krupová L, Pokorná A (2020). Quality of life in patients with non-healing wounds, with particular focus on assesment tools: A literature review. Cent. Eur. J. Nurs. Midwifery.

[3] Kus KJB, Ruiz ES (2020). Wound dressings—A practical review. Curr. Dermatol. Rep..

[4] Wang M, Zhao Q (2018). Wound dressings—A practical review. Encycl. Biomed. Eng..

[5] Mohseni M, Shamloo A, Aghababaie Z (2019). A comparative study of wound dressings loaded with silver sulfadiazine and silver nanoparticles: In vitro and in vivo evaluation. Int. J. Pharm..

[6] Yilmaz AC, Aygin D (2020). Honey dressing in wound treatment: A systematic review. Complement. Ther. Med..

[7] Hassiba AJ, El Zowalaty ME, Webster TJ (2017). Synthesis, characterization, and antimicrobial properties of novel double layer nanocomposite electrospun fibers for wound dressing applications. Int. J. Nanomed..

[8] Wilkinson LJ, White RJ, Chipman JK (2011). Silver and nanoparticles of silver in wound dressings: A review of efficacy and safety. J. Wound Care.

[9] Lemraski EG, Alibeigi S, Abbasi Z (2022). Ibuprofen@ silver loaded on poly (vinyl alcohol) / chitosan co-polymer scaffold as a novel drug delivery system. Mater. Today Commun..

[10] Mishra M, Ballal A, Rath D (2024). Colloids and surfaces B: Biointerfaces novel silver nanoparticle-antibiotic combinations as promising antibacterial and anti-biofilm candidates against multiple-antibiotic resistant ESKAPE microorganisms. Colloids Surf. B Biointerfaces.

[11] Xue CH, Chen J, Yin W (2012). Superhydrophobic conductive textiles with antibacterial property by coating fibers with silver nanoparticles. Appl. Surf. Sci..

[12] Lee SH, Jun BH (2019). Silver nanoparticles: Synthesis and application for nanomedicine. Int. J. Mol. Sci..

[13] Dong Y, Zhu H, Shen Y (2019). Antibacterial activity of silver nanoparticles of different particle size against vibrio natriegens. PLoS One.

[14] Anees Ahmad S, Sachi Das S, Khatoon A (2020). Bactericidal activity of silver nanoparticles: A mechanistic review. Mater. Sci. Energy Technol..

[15] Pavlík V, Nešporová K, Sobotka L (2021). Silver distribution in chronic wounds and the healing dynamics of chronic wounds treated with dressings containing silver and octenidine. FASEB J..

[16] Nešporová K, Pavlík V, Šafránková B (2020). Effects of wound dressings containing silver on skin and immune cells. Sci. Rep..

[17] Qin M, Liu D, Meng X (2021). Electrospun polyvinyl butyral/berberine membranes for antibacterial air filtration. Mater. Lett. X.

[18] Baskan H, Esentürk I, Dösler S (2021). Electrospun nanofibers of poly (acrylonitrile-co-itaconic acid)/silver and polyacrylonitrile/silver: In situ preparation, characterization, and antimicrobial activity. J. Ind. Text..

[19] Repanas A, Andriopoulou S, Glasmacher B (2016). The significance of electrospinning as a method to create fibrous scaffolds for biomedical engineering and drug delivery applications. J. Drug Deliv. Sci. Technol..

[20] Asghari F, Samiei M, Adibkia K (2016). Biodegradable and biocompatible polymers for tissue engineering application: A review. Artif. Cells Nanomed. Biotechnol..

[21] Tsiapla AR, Bakola V, Karagkiozaki V (2019). Biodegradable electrosprayed NPS as drug carriers for optimal treatment of orthopaedic infections. Mater. Today Proc..

[22] Nguyen DN, Clasen C, Van den Mooter G (2016). Pharmaceutical applications of electrospraying. J. Pharm. Sci..

[23] Boda SK, Li X, Xie J (2018). Electrospraying an enabling technology for pharmaceutical and biomedical applications: A review. J. Aerosol. Sci..

[24] Vilamová Z, Konvičková Z, Mikeš P (2019). Ag-AgCl nanoparticles fixation on electrospun PVA fibres: Technological concept and progress. Sci. Rep..

[25] Mochane MJ, Motsoeneng TS, Sadiku ER (2019). Morphology and properties of electrospun PCL and its composites for medical applications: A mini review. Appl. Sci..

[26] Alt Z (2022). Biointerfaces random/aligned electrospun PCL fibrous matrices with modified surface textures: Characterization and interactions with dermal fibroblasts and keratinocytes. Colloids Surf. B Biointerfaces.

[27] Ogur E (2005). Polyvinyl alcohol: Materials, processing and applications. Rapra Rev. Rep..

[28] Guarino V, Gentile G, Sorrentino L (2017). Polycaprolactone: Synthesis, properties, and applications. Encyclopedia of Polymer Science and Technology.

[29] Azimi B, Nourpanah P, Rabiee M (2014). Poly (ε-caprolactone) fiber: An overview. J. Eng. Fibers Fabr..

[30] Ghodake G, Shinde S, Kadam A (2020). Gallic acid-functionalized silver nanoparticles as colorimetric and spectrophotometric probe for detection of Al3^+^ in aqueous medium. J. Ind. Eng. Chem..

[31] Chou HL, Wu CM, Lin FD (2014). Interactions between silver nanoparticles and polyvinyl alcohol nanofibers. AIP Adv..

[32] Issa A, Al-Maadeed M, Luyt A (2017). Physico–mechanical, dielectric, and piezoelectric properties of PVDF electrospun mats containing silver nanoparticles. C.

[33] Sa B, Mukherjee S, Roy SK (2019). Effect of polymer concentration and solution pH on viscosity affecting integrity of a polysaccharide coat of compression coated tablets. Int. J. Biol. Macromol..

[34] Lee VC (2015). The antibiotic resistance crisis. P&T Peer/Rev. J. Formul. Manag..

[35] Li D, Liu Z, Yuan Y (2015). Green synthesis of gallic acid-coated silver nanoparticles with high antimicrobial activity and low cytotoxicity to normal cells. Process. Biochem..

[36] Augustine R, Kalarikkal N, Thomas S (2016). Electrospun PCL membranes incorporated with biosynthesized silver nanoparticles as antibacterial wound dressings. Appl. Nanosci..

[37] Leonas KK, Jones CR (2003). The relationship of fabric properties and bacterial filtration efficiency for selected surgical face masks. J. Text Appar. Technol. Manag..

[38] Morina E, Dotter M, Döpke C (2023). Homogeneity of needleless electrospun nanofiber mats. Nanomaterials.

[39] Yunoki S, Kohta M, Ohyabu Y (2015). In vitro parallel evaluation of antibacterial activity and cytotoxicity of commercially available silver-containing wound dressings. Plast. Surg. Nurs..

[40] Graves JL, Tajkarimi M, Cunningham Q (2015). Rapid evolution of silver nanoparticle resistance in *Escherichia coli*. Front. Genet..

[41] You C, Li Q, Wang X (2017). Silver nanoparticle loaded collagen/chitosan scaffolds promote wound healing via regulating fibroblast migration and macrophage activation. Sci. Rep..

[42] Sahu N, Soni D, Chandrashekhar B (2016). Synthesis of silver nanoparticles uusing flavonoids: Hesperidin, naringin and diosmin, and their antibacterial effects and cytotoxicity. Int. Nano Lett..

[43] Li W, Zhou J, Xu Y (2015). Study of the in vitro cytotoxicity testing of medical devices. Biomed. Rep..

[44] Siddiqui N, Asawa S, Birru B (2018). PCL-based composite scaffold matrices for tissue engineering applications. Mol. Biotechnol..

