# Supplementary material for: Silver-loaded poly(vinyl alcohol)/polycaprolactone polymer scaffold as a biocompatible antibacterial system
Source: Sci Rep. 2024 May 15;14:11093. doi: 10.1038/s41598-024-61567-5 (PMC11096175; doi:10.1038/s41598-024-61567-5)
Supplement: Supplementary file 1 — Supplementary Information. [file 41598_2024_61567_MOESM1_ESM.docx]

# Supplementary material

Silver-loaded Poly(vinyl alcohol)/Polycaprolactone Polymer Scaffold as a Biocompatible Antibacterial System

Zuzana Vilamová*^1,2^, Zuzana Šimonová^1,3^, Jiří Bednář^1^, Petr Mikeš^4^, Miroslav Cieslar^5^, Ladislav Svoboda^1^, Richard Dvorský^1,3^, Kateřina Rosenbergová^6^, Gabriela Kratošová^1^


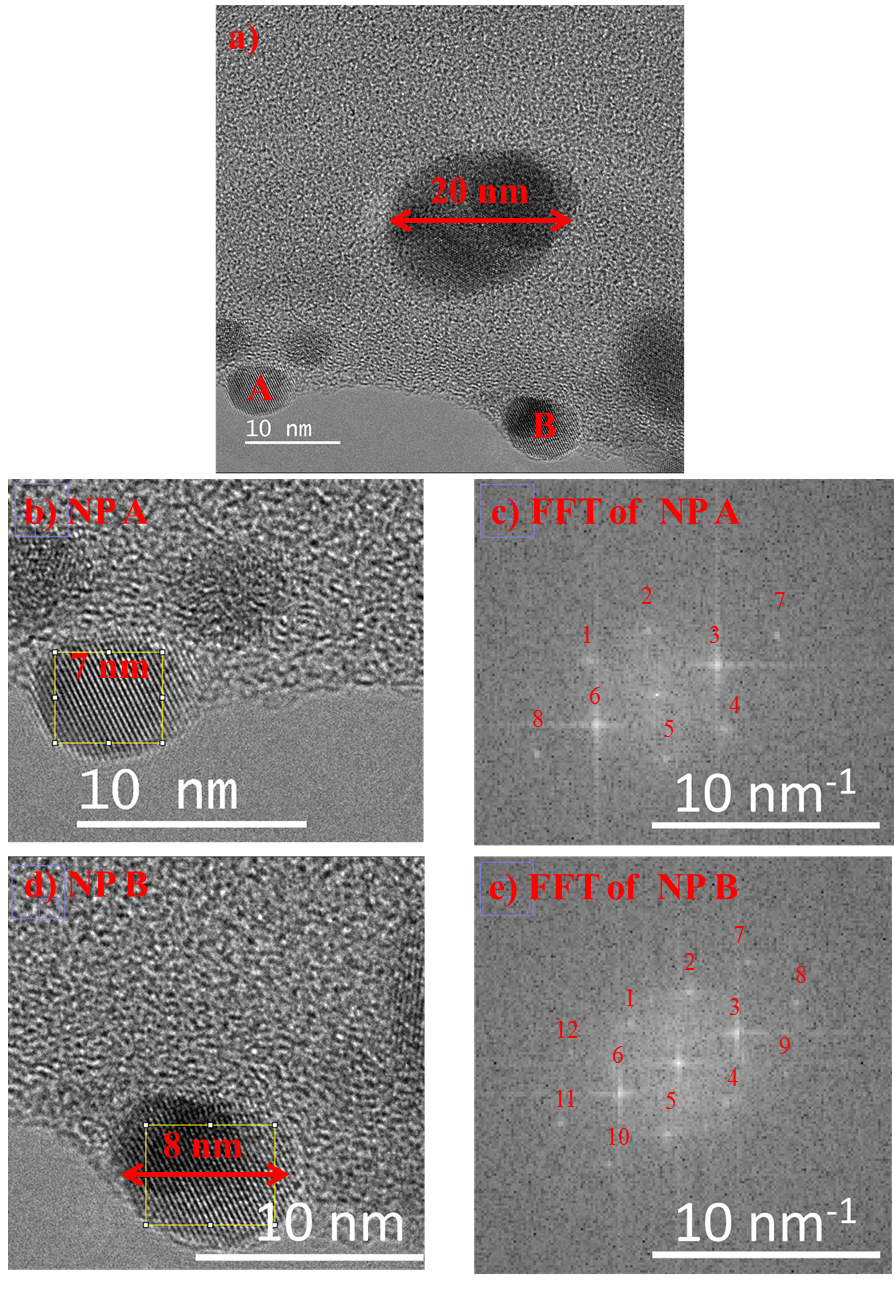


Figure S 1: HRTEM analysis of prepared a) NPs with focus on b) NP A to identify main interplanar distance of NP after application of c) Fast Fourier Transformation (FFT) in ImageJ software.

Table S 1: Measured interplanar distances of prepared nanoparticles after FFT in ImageJ software and their corresponding planes.

| **Nanoparticle** | **Point** | **d [Å]** | **{hkl}** | **Nanoparticle** | **Point** | **d [Å]** | **{hkl}** |
| --- | --- | --- | --- | --- | --- | --- | --- |
| **A** | 1 | 2.01 | 200 | **B** | 1 | 2.47 | 111 |
|  | 2 | 2.42 | 111 |  | 2 | 2.19 | 200 |
|  | 3 | 2.32 | 111 |  | 3 | 2.35 | 111 |
|  | 4 | 1.94 | 200 |  | 4 | 2.30 | 111 |
|  | 5 | 2.22 | 111 |  | 5 | 2.02 | 200 |
|  | 6 | 2.20 | 111 |  | 6 | 2.25 | 111 |
|  | 7 | 1.15 | 222 |  | 7 | 1.26 | 311 |
|  | 8 | 1.12 | 222 |  | 8 | 1.16 | 222 |
|  |  | | |  | 9 | 1.38 | 220 |
|  |  |  |  |  | 10 | 1.23 | 311 |
|  |  |  |  |  | 11 | 1.14 | 222 |
|  |  |  |  |  | 12 | 1.42 | 220 |


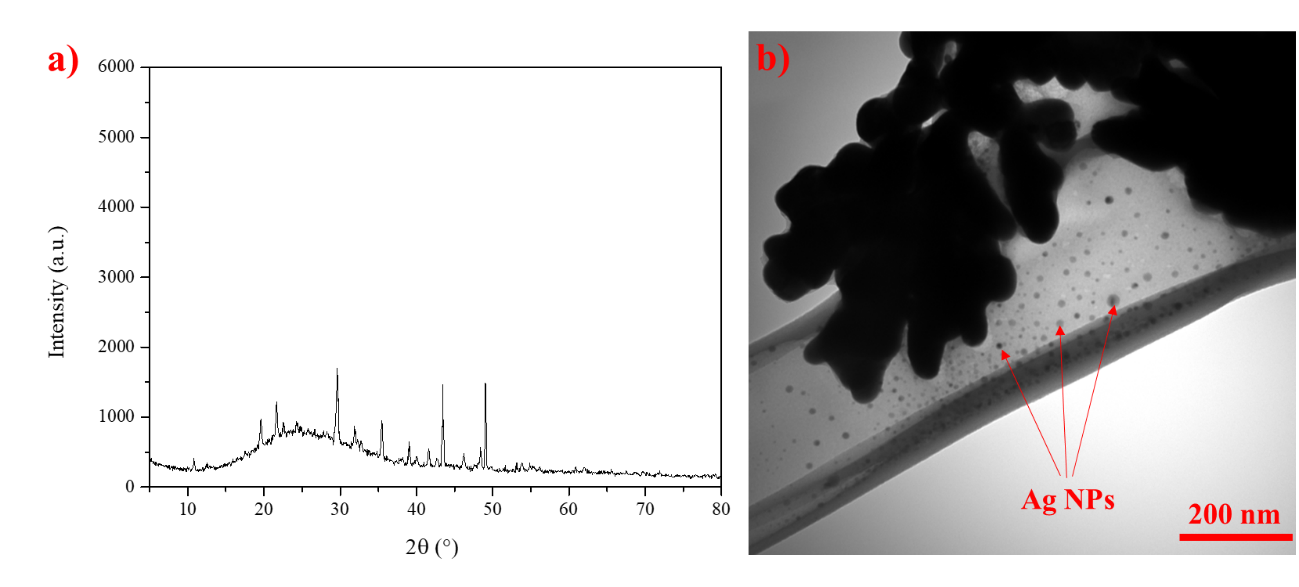


Figure S 2: a) XRD spectrum of prepared Ag NPs and AgNO_3_ solution, where peaks belong to AgNO_3_ of orthorhombic structure. B) HRTEM image of both AgNO_3_ crystal and Ag NPs.

Figure S 3: Release behavior of PCL/PVA+Ag_60 sample to DEMI water of 37 °C.
